# Undergraduate medical education amid COVID-19: a qualitative analysis of enablers and barriers to acquiring competencies in distant learning using focus groups

**DOI:** 10.1080/10872981.2021.1940765

**Published:** 2021-06-15

**Authors:** Anika Reinhart, Bastian Malzkorn, Carsten Döing, Ines Beyer, Jana Jünger, Hans Martin Bosse

**Affiliations:** a Department of General Paediatrics, Neonatology, and Paediatric Cardiology, Düsseldorf University Department (UKD), Heinrich-Heine-University, Düsseldorf, Germany; b Medical Didactics Office, Medical Faculty of the Heinrich-Heine-University, Heinrich-Heine-University, Düsseldorf, Germany; c Department of Obstetrics and Gynaecology, Düsseldorf University Department (UKD), Heinrich-Heine-University, Düsseldorf, Germany; d The German National Institute for State Examinations in Medicine, Pharmacy and Psychotherapy, Mainz, Germany

**Keywords:** COVID-19 pandemic, e-learning, clinical competence, qualitative research, mental health

## Abstract

Due to comprehensive social distancing measures related to the COVID-19 pandemic, medical faculties worldwide have made a virtue of necessity in resorting to online teaching. Medical faculties grapple with how to convey clinical competencies to students in this context. There is a need for research not only to map but also to explain the effect of these secondary measures on students’ learning and mental wellbeing. During a period of ongoing comprehensive social distancing measures in Germany, we translated a competency-based curriculum including obstetrics, paediatrics, and human genetics to an e-learning course based on online patient and teacher encounters. In our qualitative study on students’ and teachers’ views, we identify potential enablers and drivers as well as barriers and challenges to undergraduate medical education under lockdown. In summer 2020, we conducted six focus group interviews to investigate medical students’ and teachers’ perspectives, experiences and attitudes. All focus groups were videotaped, transcribed verbatim and coded. To guide our deductive and inductive analysis, we applied the theoretical framework of Regmi and Jones. Content analysis was performed in a multi-perspective group. We identified five major themes contributing to a successful use of clinical competency-based e-learning under lockdown: Communication (with teachers, students, and patients), Mental wellbeing, Structure and self-organization, Technical issues, and Learning and commitment. We discuss enablers and potential barriers within all themes and their overlap and link them in an explanatory model. In our setting, students and teachers find e-learning holds strong potential and especially in times of COVID-19 it is greatly appreciated. We broaden the understanding of the impact of distant learning on acquiring competencies, on attitudes, and on mental wellbeing. Our model may serve for a thoughtful, necessary transition to future e-learning and hybrid programs for a competency-based medical education with ongoing social distancing measures.

## Introduction

Due to the COVID-19 pandemic, the *Association of American Medical Colleges* recommended the immediate ‘*suspension on medical students’ participation in any activities that involve patient contact*’ in March 2020 [1] as medical students both are at risk and pose a risk as potential vectors due to frequent rotation and contact to staff and patients. Medical faculties all over the world were forced to switch their curricula to distant learning in virtually no time and to implement e-learning with little thoughtful evolutionary process to assure at least a minimum of learning progress of students. Particular challenges emerging are rapidly changing regulations, accountability for health of teachers and students, and need for quick technical realization [2].

Most faculties drew on elements of e-learning implemented to compensate for the loss of learning opportunities in a clinical learning environment. E-learning comprises any educational intervention mediated electronically and may complement face-to-face teaching at any degree. It provides better access to learning resources online regardless of learners’ geographical locations and timescale to enhance learning [2,3] and is embedded in medical curricula worldwide with particular experience in large territorial countries like Canada or Australia [2,4,5]. E-learning may positively complement traditional medical teaching and previous studies suggest that e-learning can be at least as effective as traditional teaching and may lead to a higher outcome regarding learning progress and competence acquisition of medical students [4,5], particularly as blended learning [3,6]. Despite growing evidence claiming e-learning may be as effective as traditional means of learning, there is a paucity of data on what actually works and how e-learning supports teaching and enhances learning [1]. Particularly, it remains uncertain whether e-learning improves or even reduces health professionals’ skills and attitudes. In their extensive Cochrane database review Vaona et al. concluded in 2016 that e-learning may make little or no difference when compared to traditional learning in patient outcomes or health professionals’ behaviours, skills or knowledge [7]: ‘*Even if e-learning could be more successful than traditional learning in particular medical education settings, general claims of it as inherently more effective than traditional learning may be misleading*’. Due to the paucity of studies and data, in their Cochrane database review involving 16 randomised trials with a total of around 5000 subjects, Vaona et al. still were unable to explore differences in effects across different subgroups [7]. In an extensive review in early 2020, Regmi and Jones identified eight factors which impact on e-learning, and assigned them to one of two categories, i.e., *enabler/drivers* and *barriers/challenges* [8]. Enablers are (i) facilitate learning, (ii) earning in practice, (iii) systematic approach to learning, and (iv) integration of e-learning into curricula. Barriers or challenges are (i) poor motivation and expectation, (ii) resource-intensive, (iii) not suitable for all disciplines/contents, and (iv) lack of IT-skills [8]. Regmi and Jones conclude that for successful integration of e-learning curriculum designers have to take into account the interaction and collaboration between learners and facilitators, to consider learners’ motivation and expectations, utilise user-friendly technology, and put learners at the centre of pedagogy.

It is likely, but an open question whether these assumptions hold true for distant or online learning in medical education under the long and comprehensive lockdown we are currently experiencing. There is a great need to ensure a comprehensive understanding of what works and why regarding potential learning opportunities in the probably ongoing predominantly distant learning [2]: How to convey specific clinical competencies exclusively online, particularly those that require clinical exposure? Which are enablers or barriers to successful acquisition of clinical competencies? How do students experience online study and social restrictions during lockdown?

In their BEME Guide to developments in response to the COVID-19 pandemic, Daniel et al. identified several publications, but only three focussing on wellbeing in undergraduate medical education [2]. The comprehensive measures of social distancing with school and university closures, restricted social connections, and loss of routine potentially have a huge negative effect on individuals’ social networks [9] and mental wellbeing [9–20] although risk factors in principle remain the same: low income, living alone, younger age, female gender, and pre-existing mental health conditions [12,13,15,21]. Thus, in addition to examining the primary impact of COVID-19, a second focus of research is needed to not only map but to explain the impact of secondary measures such as the fundamental shift of faculties to replacing or supplementing on-site teaching with e-learning on students’ learning and mental wellbeing.

In view of federal social restrictions in Germany due to COVID-19 in the summer term 2020, we translated a compulsory eight-week interdisciplinary training course for 5^th^ year medical students including obstetrics, paediatrics, and human genetics among others to an e-learning only course. In our clinical and competency-based course students contacted patients and teachers online only. In this setting, we conducted a case study among the 5^th^ year medical students and physician faculty members to explore their perspectives, experiences, feelings, and attitudes towards the online only-course to identify enablers/drivers and barriers/challenges .

## Methods

We applied a qualitative methodology within a defined theoretical framework. The strength of this approach is to consider various perspectives of a phenomenon ensuring an open in depth investigation [22]. We report study data according to the *Consolidated criteria for reporting qualitative research* (COREQ) checklist [23].

### Research team and reflexivity

#### Personal characteristics

The focus group facilitator was a senior male physician at our department with longstanding experience as facilitator of focus group discussions. The student study participants knew the moderator from two introductory online sessions of the summer term. The physician study participants knew the moderator from their work environment in our department. Both groups were informed that the aim was to explore and understand enablers and barriers of online learning in our setting and that the facilitator was part of a curriculum design group at our faculty. The curriculum design group intends to adapt and improve course concepts to meet the needs of the students and teachers.

#### Research group

We included multiple perspectives for continuing reflection of the data in our team of 3 females and 3 males from different age groups: AR is medical student, CD, HMB, and IB are senior staff and teachers from different fields of medicine, BM, HMB, JJ and IB have extensive experience in curriculum design, JJ has extensive experience in medical education policy.

### Study design

#### Context

In April 2020, facing a potential peak of COVID-19 and having to outline the upcoming term, due to federal and local regulations our medical faculty dismissed a face-to-face contact of students with patients, especially with our vulnerable population of young children, pregnant women, and the elderly patients. Nevertheless, there was the need to provide learning opportunities to progress and to reach essential certificates without abandoning the intentions of our clinical competency-based curriculum. We translated an eight-week interdisciplinary training course for N = 193 5^th^ year medical students including obstetrics, paediatrics, and human genetics among others to an e-learning only training course for the summer term 2020 with a *bring your own device* (BYOD) concept. Legally we were restricted to virtual patient and teacher encounters only, with students in live contact with patients and teachers via camera.

We defined clinical competencies that could be attained in this setting: evidence-based case management including data research and reflection. Specific competencies that potentially could not be conveyed included individual skills in physical examination and manual skills.

Key lectures (groups of 90, 24 hours) and interactive seminars (groups of 15, 34 hours) were all offered as mandatory interactive online seminars via *Microsoft Teams* (version 1.4.4.0, Microsoft Corporation, Redmond, WA, USA), including options for questions directly or through a chat during the sessions.

In each week, students worked on one long case [24] in groups of six per experienced medical teacher, commencing with taking a clinical history of a patient by the students via *Microsoft Teams* and an interactive physical examination being demonstrated by the teacher. For the long case, the teacher provided daily supervision and facilitated students defining individual PICO-questions [25,26] relating to the specific case and elaborated on them in self-directed learning and online tutorials, concluding the week with a discharge letter and a discharge summary in patient-friendly language. Specific feedback was provided during all steps of patient encounters.

For the readers’ general impression of students’ views of our course, we provide data from an anonymized and voluntary online evaluation after the course, albeit with a low response rate of N = 68 students (response rate 35%). Participating students expressed high overall satisfaction with online learning (5.2 ± 1.0, mean 5; 6-point Likert scales from 6 = very high satisfaction, 1 = very low satisfaction), with adequate workload (4.4 ± 1.2, mean 5; 6 = completely agree, 1 = completely disagree) and high learning curve (5.0 ± .9, mean 5; 6 = very high, 1 = very low).

#### Theoretical framework

We theorised that Regmi and Jones’ model (see introduction) would offer an appropriate analysis framework for our data as it creates ‘*a broader framework for making e-learning effective*’ [6] aligning potential influencers, enablers or barriers at institutional level, of facilitators and learners, delivery mechanisms, outcomes, and potential impacts in their model.

#### Research method

We chose focus groups for addressing our research question as a pragmatic tool to offer insights to participants’ attitudes, feelings, beliefs, motivation, and experiences [12] in a setting where these factors are conditional and multifaceted. The interactive and discursive character of focus group interviews seemed most appropriate these facets.

#### Sample

Participants were selected in a convenient sampling approach via e-mail, WhatsApp, social media, and face-to-face. Criteria for study participation were prior participation in our online course and participating in the study on site. All 5^th^ year medical students (N = 193) and all teachers (N = 32) of our online course were invited to participate. N = 167 students invited did not respond to the invitation to participate in the study. Fourteen of the total N = 32 teachers invited declined to take part in the study. No reasons were given.

We randomly selected N = 16 students of the N = 26 willing to participate to attend one of four focus group interviews depending on when they had time. The student participants received a small financial incentive. Students participated after completing the course when the final grades had been determined. There was no obligation or dependency in relation to any of the researchers of this study.

Accordingly, we randomly selected N = 8 teachers of the N = 18 willing to participate in one of two focus groups depending on when they had time.

#### Setting

We conducted the moderated focus group interviews in the time of tight federal and local lock down measures face-to-face on site in our department, the Department of General Paediatrics, Neonatology, and Paediatric Cardiology at the University Hospital Düsseldorf, Germany, in July 2020. Students and teachers participated separately in the focus group interviews in order to create a protected environment for the participants, particularly assuming students’ restraints in groups together with their former teachers as a confounder. Apart from the facilitator and an observer, no further persons were present during the focus group interviews.

Students’ ages ranged from 22 to 31 years (mean 24 years). Thirteen students were female (81%), three were male. Five students had a migratory background (31%). None of the students had own children. Regarding motivation for course content, four students reported personal interest in obstetrics/gynaecology (25%), five (31%) in paediatrics, and three (19%) in both, whereas three (19%) reported no specific personal interest in either field. One student had previous professional experience in nursing.

All teachers were general paediatric consultants at our department. Ages ranged from 35 years to 59 years (mean 42 years). Four were female and four male (50% each). One teacher had a migratory background. Five of the participants had own children (63%). Teachers’ prior clinical experience as measured by work years ranged from 1 year to 30 years (mean 11 years). Self-assessed experience with e-learning was within a wide range from virtually no prior experience (N = 2) to very experienced (N = 2). Sample characteristics are depicted in Table 1.

**Table 1.** Sample characteristics

	5^th^ year medical students	Teachers
	N = 16	N = 8
**Gender**		
female	13 (81%)	4 (50%)
male	3 (19%)	4 (50%)
**Age** (in years)		
minimum	22	35
maximum	31	59
mean	24	42
**Migration background**		
Yes	5 (31%)	1 (12.5%)
No	11 (69%)	7 (87.5%)
**Teachers’ postgraduate training (in years)**		
minimum		1
maximum		30
median		11
**Teachers’ e-learning experience (in years)**		
minimum		1
maximum		5
mean		2,9
**Students’ nurse training**		
yes	1 (6%)	
no	15 (94%)	
**Own children**		
yes	0	5 (62.5%)
no	16 (100%)	3 (37.5%)
**Students’ personal interest**		
Pediatrics	9 (56%)	
Gynecology	7 (44%)	
Geriatrics	0	
None of these	3 (19%)	

To include multiple perspectives, we show participants’ heterogeneous backgrounds on age, gender, migration background, own children, prior professional training, and (for students) interest in the specific fields.

#### Data collection

The six focus group discussions lasted between 60 and 90 minutes each. Discussion was prompted using a questioning route with open-ended questions defined for both students’ and teachers’ focus group interviews (Supplement 1). The facilitator encouraged contribution of all participants. Group discussions were videotaped, pseudonymised, and transcribed verbatim [22]. All study participants consented to the recording of the focus group interviews. We chose video recordings as the mouth-nose protection worn during the study made matching difficult otherwise. The video recordings also allowed including non-verbal language in our analysis. Field notes were made during and after each focus group session by the facilitator and an observer. Participants were later provided with the results, not the transcripts for correction.

### Analysis and findings

#### Data analysis

Regmi and Jones’ model served as an appropriate content analysis framework for a deductive approach to our focus group data. New codes were added in an inductive manner in an iterative process as described earlier [16]. Two researchers (AR and HMB) coded data independently. Results of our analysis were combined with behavioural data (observations of non-verbal communication in the videos in case of emotional topics) for deeper insights. We found saturation of arguments in the last of four students’ and two teachers’ focus groups, respectively. Any coding disagreements were discussed, settled, and adjusted accordingly. Emerging codes were attributed to categories and categories to broader themes in a hierarchical coding frame. Data was then reflected, discussed and visualised in the whole research group. In their approval of the results, participants added no further categories or themes.

#### Reporting

Data are presented according to the categories and major themes that emerged from our findings. We include illustrative pseudonymised quotations of students and teachers. Pseudonymisation numbers refer to the focus group (first digit) and the individual within the respective focus group (second digit). S = student and T = teacher. We found consistency between the quotations and our findings throughout our results. Emotional quotes were consistent with non-verbal language.

All codes were assigned to emerging categories and major themes (see results section, five themes: *Mental wellbeing, Communication, Structure and self-organization, Technical issues, Learning and commitment*). We use the term ‘mental wellbeing’ in the comprehensive sense of the WHO definition [27].

We present the major themes and link them with their respective overlaps (as minor themes) to an explanatory model.

## Results

**Figure 1.**
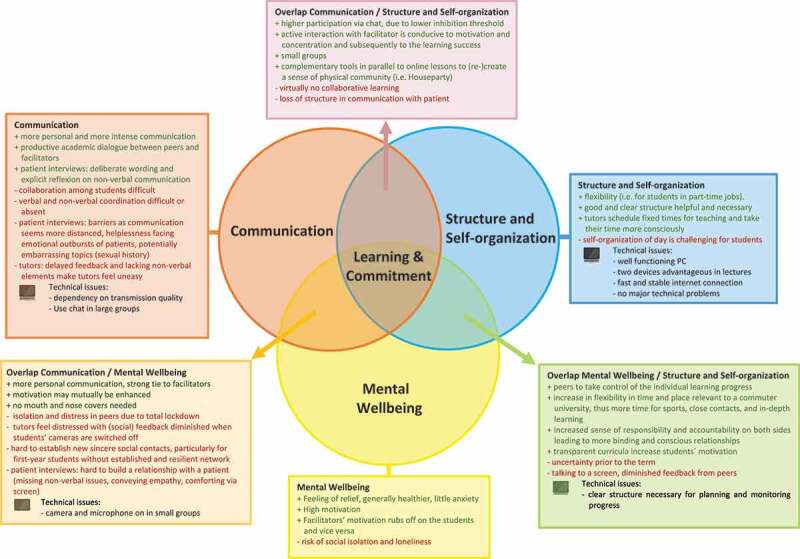
Conceptual framework of themes with impact on e-learning in health sciences education

Five major themes emerged from the four focus groups with students and the two focus groups with medical teachers: *Mental wellbeing, Communication* (with teachers, students, and patients), *Structure and self-organization, Technical issues*, and *Learning and commitment*. All contributed to a potentially successful use of e-learning in the setting of a lockdown. We present the five emerging themes and characteristic quotations of students and teachers in their focus group discussions. We aligned these five themes including their overlap to an explicatory model in a Venn diagram [28] (Figure 1). The eight factors identified as enablers or as barriers within the framework of Regmi and Jones may be assigned to three of these five themes (Table 2 [8];). In addition to their framework, we identified the themes *Technical issues* and *Mental wellbeing*.

**Table 2.** Themes contributing to successful e-learning

Five themes of the explanatory model (Figure 1) vs. enablers and barriers of Regmi and Jones’	1. **Facilitate learning**	2. **Learning in practice**	3. **Systematic approach to learning**	4. **Integration of e-learning into curricula**	5. **Poor motivation and expectation**	6. **Resource-intensive**	7. **Not suitable for all disciplines/contents**	8. **Lack of IT skills**
Theme 1. **Mental wellbeing**								
Theme 2. **Communication** Among students Between students and tutor Between students and patients	X							
Theme 3. **Structure and self-organization**			X	X		X	X	
Theme 4. **Technical issues**								X
Theme 5. **Learning and commitment**		X	X		X			

We relate the five themes of our explanatory model (left column) to eight separate themes of Regmi and Jones’ model describing enablers (themes 1 to 4) and barriers (themes 5 to 8) [8]. Our theme *Mental wellbeing* is not represented in Regmi and Jones’ model.

Conceptual framework of themes with impact on e-learning in health sciences education. Venn diagram [28] depicting the five themes *Mental wellbeing, Communication, Structure and self-organization, Technical issues*, and – at the centre of the model – *Learning and commitment*. Enablers (green font) and barriers (red font) of the themes *Mental wellbeing* (yellow), *Communication* (red), *Structure and self-organization* (blue), *Technical issues* (PC icon) as well as their overlap are depicted. Theme *Learning and commitment* is the centre of the model.

### Non-verbal communication

Study participants used gestures and facial expressions to underline what they literally say. The only incongruence of verbal and non-verbal communication we found was related to mental wellbeing, when participants partially seemed to searching for the appropriate wording. This in turn may show that this aspect is emotionally important. In following up these scenarios, we had the impression that with our open-ended questions and time for reflection all aspects of this aspect were finally addressed in each focus group. Our findings on non-verbal communication are detailed in Supplement 2.

### Theme 1. Mental wellbeing

Both students and by teachers welcome the distant learning semester with relief as a successful alternative. It all seems new and ‘*refreshing*’ (4.4 S). The stress level of both groups is relatively low, if initial (mainly technical) barriers are overcome. The motivation of the students and teachers is high and may be increased mutually: that of the students by active, motivated teachers addressing them personally by name and that of the teachers by a high participation of the students.

Most students generally feel healthier, less stressed, and engage more in sports. Students are more flexible in time and space and can put more focus on their selves and spend time with their family or partner. The perceived learning requirements are considered comparable to previous face-to-face semesters. Negative effects on wellbeing are prolonged screen time and the uncertainty prior to the term whether ‘everything will work out well’ (6.6 S). The students miss social contact with their fellow students, as they do not have common breaks or collaborative learning sessions. This may result in loneliness. In addition, online teaching may become monotonous in some cases, which has a negative effect on motivation.

An online semester is easier when social relationships are already well established. Students then may draw from established social resources, as opposed for first-year students. Spending time with fellow students on campus, 5^th^ year students consider their previous face-to-face study time prior to the COVID-19 pandemic the ‘*best time in our studies*’ (2.2 S) in which social contacts could be established for life.

For teachers, the online semester is rather more relaxed as they wear a mouth-nose-protection during their clinical routine in times of the COVID-19 pandemic and can take it off during teaching sessions which they hold in a separate room. Teachers sometimes feel lonely facing the camera, and they miss the personal interaction with the students. For them, preparation and online teaching is more strenuous (i.e., by organizing patients in time) and requires greater concentration during the lessons.

Students and teachers enjoy the flexibility in terms of time and above all place, and the overall atmosphere is more relaxed. Working in flexible locations saves on travel and allows students to spend time with their family back home and/or their partner, as they can join in from anywhere. Teachers enjoy the fact that they can move appointments flexibly, partly since no physical room is required.

### Theme 2. Communication

#### Communication among students

According to students, there is less and in part more distanced communication during the online term. Time spent together during lectures, breaks or in the library, for example, is missing. Collaboration is more difficult online although some advantages are seen. Students in part-time jobs have the opportunity to communicate independent of location. Communication via WhatsApp is common but may lead to delay until the other person is online.

Students use complementary programs for communication enabling them to clarify questions regarding course content as well as organisational matters. Our course was structured with Microsoft Teams. Students mainly use WhatsApp in their communication regarding learning issues. Sessions of Houseparty, an app that facilitates group video calls, in parallel to online lessons (re-)create a sense of physical community. Students do not see any problem to access medical content and private information on one end device.

Student: This [Houseparty] restores a little of what is otherwise lost, so if something funny happens in the lecture, something funny is said, then we laugh about it together and so we could laugh or make a comment together. (4.2 S)

#### Communication between students and teacher

The communication with the teacher is perceived as more intensive and more connected by both students and teachers, as all explicitly take their time. Specific measures may enhance productive communication between teachers and students: a personal first-name approach, the motivation of the teacher, all switching their cameras on, and a complementary chat function. A clear allocation of students to a teacher increases commitment and the teacher´s sense of responsibility.

A ‘real’ teacher, albeit via camera, provides a more personal communication. An active teacher interaction is conducive to motivation, concentration, and subsequently to the learning success. An active interaction with the teacher fosters students setting priorities and filtering important learning content. It also helps students check on their learning success. It promotes attentive listening and students’ participation and activity.

Face-to-face communication with cameras switched on fosters connectivity on both sides. On the students’ side, this is mainly due to the close face resolution of the teacher via camera. Teachers perceive the students less intensively if their camera is switched off which is due to the high inhibition threshold for the camera among the students.

Student: “I felt closer to the lecturers this semester. I think the reason was that you could see the face in full size with all facial expressions while he was giving the lecture.“ (1.2 S)

Students receive more and more direct feedback from the teachers in patient contact, which is highly appreciated by the students.

Teacher: “I have the feeling that if you teach on the run in the everyday life of a ward you are simply too brusque, and conversations are interrupted because you get paged or whatever. You simply take your time because you say: Okay, I have an hour of teaching now, lock myself in my room with patient and camera. You then really take focussed time for the [students].” (3.3 T)

In contrast, without visual feedback from students with cameras switched off it is difficult for teachers to assess whether and how well the students follow the course, to determine who is focused, and to monitor the students’ learning progress leading to high unease among the teachers.

Teacher: I simply thought it was much more strenuous in terms of concentration […]. There is no feedback at all. […] You can’t estimate: Do they think it’s good or not so good right now? Are they rather bored or do they follow? Particularly, I found that very difficult.(3.4 T)

For fostering medical competencies, small groups are pivotal because it makes communication easier and more direct. When students already feel familiar with each other they are more comfortable to speak up in the group, give answers or ask questions.

In large groups, complementary chat-features lower psychological barriers to participation, which is experienced generally higher by students and teachers during the online semester.

Student: I think I noted as pro that the chat function was very pleasant. It simply lowered the inhibition threshold and I think more people were more active. (1.3 S)

Teachers then may serve as a role model even in an online-only term, but rather as a teacher and not as a physician in his or her daily work routine.

#### Communication between students and patients

Students can communicate with patients and take a medical history in the online format. Theoretical facts can be collected, but the following things may be missing in the online communication with patients according to students: the first general impression, non-verbal aspects, body posture, and the patient’s personal environment so that a holistic picture may not evolve.

Speaking to the patient online may be more intense as a more conscious attention is paid to language and specific wording.

Student: We also had to do a lot of verbal stuff that otherwise charisma and facial expression might have done. […] What I mean by communication and empathy, that you choose your words much more consciously to express empathy.(2.1 S)

Barriers are interviews in a group of several students, transmission quality, patient uneasy with speaking to a camera, language barriers, hearing loss or patients unfamiliar with technology or lacking technical devices. For a successful acquisition of competence in taking a medical history, it is important to have clear rules on the procedure and content.

Student: I gained a lot in my competency relating to empathy, because I found it very difficult to show empathy on a screen to a patient while taking a history. It was really hard and then you were much more conscious in your communication with the patient. […] Because you don’t have all the gestures and facial expressions. (2.3 S)

It is more difficult to establish a relationship with the patient and to get to know each other via a camera. Students view building their relationship with the patient more strenuous in the online format because they experience a higher inhibition threshold to ask personal questions (e.g., sexual history). Non-verbal aspects are lost via the camera and spontaneous reactions are difficult or missing. A longitudinal patient contact or one-to-one communication would alleviate these barriers. Students feel particularly helpless facing emotional outbursts of patients in online interviews. Teachers believe that building a relationship with the patient is essential for future patient-physician relationships and fear that this can only be established to a limited extent in online format. The camera to be switched on is considered essential by teachers in the communication with students and patients.

Teacher: In conservative medicine, building relationship is the absolute be-all and end-all in many issues. This applies to all general practitioners and paediatricians, neurologists and gynaecologists and so on, where much talking medicine is paramount, and everything only works through building a relationship. Of course, this was only possible to a very limited extent in this setting. (3.1 T)

### Theme 3. Structure and self-organization

Both students and teachers consider a good and clear structure including a timetable to be helpful and necessary to organize their learning content and to take control of their learning progress. Clear work assignments and a transparent curriculum are appreciated on both sides. The personal time management is challenging but also a good training for the students to organize themselves.

Teacher: Now I had one and a half hours in my calendar for the students and nothing else. In the past setting, you do your work and go like: Oh, there’s the student. Now I have to devote another ten minutes to him. Now time was specifically reserved for them. And that makes working productive, of course. (5.4 T)

Student: The lecturers have now consciously taken specific time for us. [They] really had time to answer our questions, to discuss this with us.(2.2 S)

### Theme 4. Technical issues

There were no major technical problems. If at all, teachers have more technical problems than students do when participating in the online semester. As for students in our setting, we found no significant general technical problems related to soft- or hardware or to IT-skills in general.

Hardware: A well-functioning laptop is a prerequisite for participation in the online term. The use of a second device or a split screen offers a good overview during the course and taking detailed notes in parallel.

Software: After overcoming initial difficulties, Microsoft Teams can be used intuitively and is a suitable platform for the online semester. There should be only one e-learning platform in use. An unambiguous ID of students when registering for a course is mandatory.

A fast and stable internet connection is required on both sides. Time delay restricts interactivity and flow. An unstable internet connection leads to time lags in communication, which makes it difficult to ask interposed questions.

### Theme 5. Learning and commitment

From students’ and teachers’ perspective, distant learning may have benefits on acquiring specialized and applied knowledge, and thus in this respect the general learning success may be higher. Learner-centred work may be promoted in an online term by fostering individual pace in learning, but barriers are described i.e., limits to setting own preferences. There has hardly been any collaborative learning during the online semester. Students do not work on their common tasks together but rather divide tasks among them.

Online learning fosters deep learning at the expense of acquiring manual competencies due to lack of deliberate practice. In depth learning is fostered by reducing patient contacts and students focussing on deeper information sources and primary literature and specific guidelines instead of summaries and superficial search engines. Students hardly read any textbooks during the online term.

The learning style of the students subjectively remains more or less the same during the online semester. Particularly in higher semesters, students have usually found their individual learning style already.

The competencies acquired in the online term include case management, data acquisition, personal organisation, and managing personal time as well as drafting medical reports and structuring referrals.

Students dearly miss practical training in the distant learning semester. Specific and relevant competencies that were not acquired include physical examination and essential manual skills. This is perceived as handicap for the future personal professional career. Furthermore, the virtual patient contact only may be suggestive to students that the physical examination generally is of limited importance – which is seen as an erroneous trend from teachers’ perspective.

Student: I think [physical examination] is important that this is not omitted, because the question: How do I establish relationship with a patient? Which instructions do I use? How do I touch him? I think that’s something that is very helpful if you have done it a few times before going to the department.(1.2 S)

Students do not physically experience their future work environment – which teachers and students alike regard as essential for a student’s personal orientation of their future focus of training.

## Discussion

In this case study, we investigated students’ and teachers’ views on the implementation of e-learning in a course entirely delivered as distant learning, identifying enablers and barriers. In our setting, thoughtful and attentive communication, generally high mental wellbeing, and alignment of content and assessment all contributed to a high motivation and commitment of both students and teachers averting potential barriers previously described [8,12,13,15,29–31].

In 2018, Vaona et al. [2] found just 16 randomised trials addressing the specific impact of e-learning on relevant outcomes in medical education: patient outcomes or health professionals’ behaviours, skills or knowledge. Our study does not address these outcomes but rather examines students’ and teachers’ views on e-learning in our setting. We model five emerging themes including their overlap (Figure 1) explaining for their interrelationship: *Mental wellbeing, Communication, Structure and self-organization, Technical issues*, and *Learning and commitment*. This may guide curriculum planners on how to adapt programs for competency-based medical education under such strict social distancing restrictions – and to avoid potential adverse effects.

Generally, both health educators and students addressed a potentially positive impact of e-learning in our setting on acquiring knowledge. Students utilize various electronic databases but virtually no textbooks in line with prior findings [29]. Skills were acquired as competencies in case management, data acquisition, personal organisation, and managing personal time, as well as in drafting medical reports, and structuring referrals, in line with prior findings [32]. Relevant competencies not acquired in our setting included physical examination and manual skills although findings suggest that some manual skills may be acquired at home with a feedback of faculty staff on videotaped procedures [33]. A major challenge for students is the need for self-discipline to practice autonomously. Our heterogeneous findings regarding the acquisition of clinical competencies may explain why Vaona et al. remain unsure whether e-learning improves or even reduces health professionals’ skills [7].

Beyond our setting, students and teachers both state that e-learning as distant learning may enhance course participation, foster deepening of medical issues, provide an enormous flexibility in time and space with more time to learn, and provide a strong(er) tie to the teacher. In their view may have a potential positive impact on in-depth learning and both students’ and teachers’ mental wellbeing. Our findings are in line with Daniel et al.’s BEME Guide [2]: *good in theory, but challenging in practice*.

In addition to publications to date, we have seen that e-learning as distant learning in our setting could affect future health professionals’ attitudes, which Vaona et al. do not address. Potential barriers in communication via camera in an online setting make students speak more consciously to their patients and select their words more deliberately. In its limitations to communicate via camera, they discover the value of non-verbal communication for building relationships and in understanding the patient’s perspective.

We applied the framework of Regmi and Jones [8] for our analysis, who describe four factors explaining successful e-learning: (i) fostering communication and collaboration between learners and facilitators, (ii) considering learners’ motivation and expectations, (iii) utilising user-friendly technology, and (iv) putting learners at the centre of pedagogy. Our main factors generally intersect with this model. We expand Regmi and Jones’ factor (i) communication in specifying issues relating to communication among students (as a base for collaborative learning), and we add communication with patients as a sub-factor, which Regmi and Jones did not consider. We do see a very high motivation of both students and teachers in the setting of medical education during social distancing – which may explain why this does not emerge as an independent factor in our findings. Unsurprisingly, factor (iii) outlines feasible and acceptable technological solutions as a prerequisite in our findings. In contrast to prior publications [8,29,34,35], we found no significant general technical problems related to soft- or hardware or to IT-skills in general (see Figure 1). Learners should be at the centre of all didactical measures as a fourth factor (iv), corresponding to our factor Learning and commitment.

### Mental wellbeing

Regmi and Jones [8] do not address our theme *Mental wellbeing* in their model. Probably this issue surfaces to date due to the strict social distancing regulations and the lack of embedding e-learning in blended learning scenarios. Curriculum planners need to observe this new emerging issue in medical education under social distancing and to develop interventions and preventive strategies [19].

Generally, the online term is viewed as a ‘*refreshing change*’ (4.4 S) and met with relief. The motivation of students and of teachers is high and rubs off on each other. Both generally feel healthier and less stressed. In overlap of the themes *Mental wellbeing* and *Structure and self-organization* this may be explained on the one hand by an increase in flexibility in time and place relevant to a commuter university as ours. This provides more time for sports and close contacts and on the other hand by an increased sense of responsibility and accountability on both sides leading to a more binding relationship. As a result students commit to learn at their own pace and location [8,36]. From students’ and teachers’ views, all these factors may foster in-depth learning and lead to a high satisfaction with the personal learning success. In overlap of the two themes *Mental* w*ellbeing* and *Communication*, enablers are a more personal communication with the teachers perceived by students. A significant barrier to wellbeing may be the feeling of isolation in line with prior findings [9,29,31]. The students’ statements suggest that this may be reinforced by the reduced communication among students.

In our study, students report that they miss social contact to their fellow students, which has also been shown by subsequent studies [9,19]. Lockdown measures and social constraints in times of COVID-19 may lead to an increased feeling of loneliness and depressive symptoms [9,13,16,19], which is not solely due to e-learning but may affect its outcomes. With mental wellbeing and learning behaviours intricately interlinked, COVID-19 may thus lead to a decrease in learning outcomes [18]. To compensate for these drawbacks, future online curricula need to stress and support the potentialflexibility in time, pace and space, to foster binding relationships between students and teachers through mindful communication and longitudinal contacts, and to be transparently structured for learners to monitor their learning progress as a prerequisite for their motivation.

### Communication

Students and teachers generally feel closer and more connected to each other in an online format – fostered by the common goal of a successful online semester during a lockdown. Their communication is more personal and more intense, as all explicitly take their time. Teachers’ express a greater sense of responsibility and prepare their sessions more specifically. A ‘real’ teacher (albeit via camera) provides a more personal communication, calls for increased activity [37], and their motivation rubs off on each other. Students and teachers report that small groups of students being supervised by a teacher can improve communication which may lead to an increased learning outcome [33].

Students’ communication with ‘real’ patients may be realized via camera in principle to make learning effective [35], yet results in a more distanced relationship with patients. Online communication between students and patients is possible when technical requirements, such as a stable internet connection, a working camera and microphone are met. Students and teachers view students’ communication with patients comparatively more disrupted than with (fellow) students and teachers. Particularly, both are sceptical that e-learning may not adequately foster building a relationship with a patient which they see at the centre of a patient-physician relationship. Significant barriers are (i) from the students’ view interviews in groups and addressing potentially sensitive topics such as a sexual history, (ii) from the patient’s viewpoint feeling uneasy to speak via camera and language barriers, and (iii) regarding technical issues, poor transmission quality. This contrasts with findings of Regmi and Jones – probably due to our online-only approach excluding the necessary deliberate practice for consolidation of such competencies. We see a positive impact on students’ attitudes on their deliberate wording and on reflecting on non-verbal communication. Such an element of reflection should be part of future curricula. Conversely, students feel particularly helpless facing emotional outbursts of patients in online interviews. Clear guidelines to structure the interview and a longitudinal liaison are therefore needed in future.

Generally, students are well connected online with social media, in our setting mostly in dedicated groups in WhatsApp. Online-only courses pose a threat of social isolation [9]. There has been early recommendation to look after others and oneself to improve social distancing [38]. Students assume that in their early academic years freshmen may be more prone to such social isolation than those in higher years in line with prior findings [13], as the latter may have already established lasting and resilient social contacts. Students find measures to alleviate potential social isolation, i.e., in using complementary channels to create the feeling of togetherness (i.e., the face-to-face-network Houseparty in parallel to online lectures on Microsoft Teams). The relevance of social contacts for students’ mental wellbeing during the COVID-19 pandemic is well established and in future, there is a need to identify and support students at higher risk of negative psychological effects [9,11,14].

### Limitations

In this study we address students’ and teachers’ views and do not answer the specific impact of e-learning on patient outcomes or health professionals’ behaviours, skills or knowledge [2], so immediate conclusions for future curricula need to be drawn with caution. Nature and content of our course was adapted significantly for distant learning. Then again, we do not make comparisons to past or future courses, but by translating students’ and teachers’ views on distant learning to a model we offer guidance to faculty staff on thoughtful development of future curricula under distant learning. We do elucidate the complex interrelationship of underlying factors in our setting to explain perceived enablers and barriers to the processes of acquiring competencies. We took great care to reflect on potential confounders in this study. The sampling procedure with a large number of students not responding to our invitation may have led to a bias regarding the breadth and depth of perspectives. Nevertheless, our study participants vary in terms of age, gender, migration background, and personal interests. We intended to avoid a potential social-desirability bias regarding students’ contributions and therefore conducted the focus groups after students had concluded the course. As part of our faculty, we naturally cannot completely exclude this point. The focus group facilitator was male potentially inducing a gender bias. We specifically and reiteratively reflected on such issues from multiple perspectives in our (heterogeneous) research group.

The setting of comprehensive social distancing in this summer term was an extreme and novel situation, which may not compare to past settings. Both students and teachers were highly relieved that an online-only course was offered as bridging which may be a potential confounder. Our findings are not generalizable regarding e-learning as such, but they do give curriculum designers support to understand the impact on students and teachers of current courses under a lasting lockdown. It therefore remains to be seen whether these experiences apply one-to-one onto future curricula carefully re-implementing clinical experiences and assessments that can only be met through direct patient contact [39]. It is also unclear whether the perceived positive effects of this term are sustainable. We did not specifically address future health professionals’ performance which e-learning may foster [8,40], and the scope of our study cannot include patient outcomes. Data is limited on both issues, particularly relating to sustainability of effects [41].

### Conclusions

In conclusion, e-learning holds strong potential and especially in times of COVID-19 with ‘*disrupted training*’ [42] both students and teachers full of appreciation. At least temporarily, an online-only design of an entire curriculum seems acceptable although there is no dispute that digital patient encounters should generally remain a makeshift solution. This setting may enhance engagement, provide an enormous flexibility in time and space resulting in more time to learn, provide a strong(er) tie to teachers and thus may have a potential positive impact on learning autonomy, and both students’ and teachers’ mental wellbeing.

A number of publications have raised the important and evident issue of mental wellbeing among (medical) students under Covid19 in general. To our knowledge, we are the first to provide a differential view on specific enablers and barriers to mental wellbeing resulting from distant learning as such.

Furthermore, we are the first to address a positive impact on specific future health professionals’ *attitudes*: Facing potential barriers in communication, students value non-verbal communication for understanding the patient’s perspective and building relationships, and they generally reflect on their communication more consciously. Such a (positive) impact of distant learning on attitudes of future graduates has not been addressed so far.

Major drawback is the potential social isolation with virtually no collaborative learning especially for groups without a resilient social network. Additionally, e-learning may not adequately foster building a relationship with a patient and students have no opportunity to explore potential future work environments.

We contribute to understanding the impact of distant learning on acquiring competencies, on attitudes of future graduates, and on their mental wellbeing relating to distant learning. Our results may offer guidance on the thoughtful design of future (medical) curricula for a competency-based medical education integrating distant learning – and at the same time alleviate its potentially adverse effects on learning and mental wellbeing. Our model may support a thoughtful, necessary transition to future e-learning and hybrid programs to advance medical education beyond the COVID-19 pandemic.

## Supplementary Material

Supplemental Material

## Trial registration

This study was registered in the German Clinical Trials Register, DRKS (DRKS00022251).

## References

[1] Whelan A, Prescott J, Young G, et al. Association of American Medical Colleges: guidance on Medical Students’ clinical participation: effective immediately. Washington DC 2020.

[2] Daniel M, Gordon M, Patricio M, et al. An update on developments in medical education in response to the COVID-19 pandemic: a BEME scoping review: BEME guide no. 64.Med Teach. 2021;43(3)1–13. 10.1080/0142159X.2020.1864310 33496628

[3] Garrison DR, Kanuka H. Blended learning: uncovering its transformative potential in higher education. Internet Higher Educ. 2004;7:95–105.

[4] Munro V, Morello A, Oster C, et al. E-learning for self-management support: introducing blended learning for graduate students - a cohort study. BMC Med Educ. 2018;18(1):219. . 30249238 10.1186/s12909-018-1328-6 PMC6154791

[5] Salajegheh A, Jahangiri A, Dolan-Evans E, et al. A combination of traditional learning and e-learning can be more effective on radiological interpretation skills in medical students: a pre- and post-intervention study. BMC Med Educ. 2016;16(1):46. 26842495 10.1186/s12909-016-0569-5 PMC4739398

[6] Liu Q, Peng W, Zhang F, et al. The effectiveness of blended learning in health professions: systematic review and Meta-Analysis. J Med Internet Res. 2016;18(1):e2. 26729058 10.2196/jmir.4807 PMC4717286

[7] Vaona A, Banzi R, Kwag KH, et al. E-learning for health professionals. Cochrane Database Syst Rev. 2018;1:CD011736. 29355907 10.1002/14651858.CD011736.pub2 PMC6491176

[8] Regmi K, Jones L. A systematic review of the factors - enablers and barriers - affecting e-learning in health sciences education. BMC Med Educ. 2020;20(1):91. 32228560 10.1186/s12909-020-02007-6 PMC7106784

[9] Elmer T, Mepham K, Stadtfeld C. Students under lockdown: comparisons of students’ social networks and mental health before and during the COVID-19 crisis in Switzerland. PloS One. 2020;15(7):e0236337. 32702065 10.1371/journal.pone.0236337 PMC7377438

[10] Alsoufi A, Alsuyihili A, Msherghi A, et al. Impact of the COVID-19 pandemic on medical education: medical students’ knowledge, attitudes, and practices regarding electronic learning. PloS One. 2020;15(11):e0242905. . 33237962 10.1371/journal.pone.0242905 PMC7688124

[11] Cao W, Fang Z, Hou G, et al. The psychological impact of the COVID-19 epidemic on college students in China. Psychiatry Res. 2020;287:112934. 32229390 10.1016/j.psychres.2020.112934 PMC7102633

[12] Dodd RH, Dadaczynski K, Okan O, et al. Psychological Wellbeing and Academic Experience of University Students in Australia during COVID-19. Int J Environ Res Public Health. 2021;18(3):866. 10.3390/ijerph18030866 PMC7908219 33498376

[13] Essangri H, Sabir M, Benkabbou A, et al. Predictive factors for impaired mental health among medical students during the early stage of the COVID-19 pandemic in Morocco. Am J Trop Med Hyg. 2021;104(1):95–102. . 33205748 10.4269/ajtmh.20-1302 PMC7790070

[14] Guse J, Heinen I, Kurre J, et al. Perception of the study situation and mental burden during the COVID-19 pandemic among undergraduate medical students with and without mentoring. GMS J Med Educ. 2020;37(7):Doc72. 33364351 10.3205/zma001365 PMC7740010

[15] Jacobs R, Lanspa M, Kane M, et al. Predictors of emotional wellbeing in osteopathic medical students in a COVID-19 world. J Osteopath Med. 2021;121(5):455–461. 10.1515/jom-2020-0272 33694347

[16] Kalok A, Sharip S, Abdul Hafizz AM, et al. The Psychological impact of movement restriction during the COVID-19 outbreak on clinical undergraduates: a cross-Sectional study. Int J Environ Res Public Health. 2020;17(22):22. 10.3390/ijerph17228522 PMC7698578 33212969

[17] Lyons Z, Wilcox H, Leung L, et al. COVID-19 and the mental well-being of Australian medical students: impact, concerns and coping strategies used. Australas Psychiatry. 2020;28(6):649–652. 32772729 10.1177/1039856220947945 PMC7424607

[18] Meo SA, Abukhalaf AA, Alomar AA, et al. COVID-19 pandemic: impact of quarantine on medical students’ mental wellbeing and learning behaviors. Pak J Med Sci. 2020;36:S43–S8. [COVID19-S4]. 32582313 10.12669/pjms.36.COVID19-S4.2809 PMC7306952

[19] Son C, Hegde S, Smith A, et al. Effects of COVID-19 on college students’ mental health in the USA: interview survey study. J Med Internet Res. 2020;22(9): e21279. 10.2196/21279. 32805704 10.2196/21279 PMC7473764

[20] Yang KH, Wang L, Liu H, et al. Impact of coronavirus disease 2019 on the mental health of university students in Sichuan Province, China: an online cross-sectional study. Int J Ment Health Nurs. 2021. DOI:10.1111/inm.12828 PMC8242514 33704896

[21] Grubic N, Badovinac S, Johri AM. Student mental health in the midst of the COVID-19 pandemic: a call for further research and immediate solutions. Int J Soc Psychiatry. 2020;66(5):517–518. 32364039 10.1177/0020764020925108 PMC7405631

[22] Krueger RACM. Focus groups: a practical guide for applied research. 5th ed. Los Angeles, CA: Sage Publications;2015.

[23] Tong A, Sainsbury P, Craig J. Consolidated criteria for reporting qualitative research (COREQ): a 32-item checklist for interviews and focus groups. Int J Qual Health Care. 2007;19(6):349–357. 17872937 10.1093/intqhc/mzm042

[24] Thistlethwaite JE, Davies D, Ekeocha S, et al. The effectiveness of case-based learning in health professional education. A BEME systematic review: BEME guide no. 23. Med Teach. 2012;34(6):e421–44. . 22578051 10.3109/0142159X.2012.680939

[25] Richardson WS, Wilson MC, Nishikawa J, et al. The well-built clinical question: a key to evidence-based decisions. ACP J Club. 1995;123(3):A12–3. 10.7326/ACPJC-1995-123-3-A12 7582737

[26] Eriksen MB, Frandsen TF. The impact of patient, intervention, comparison, outcome (PICO) as a search strategy tool on literature search quality: a systematic review. J Med Libr Assoc. 2018;106(4):420–431. 30271283 10.5195/jmla.2018.345 PMC6148624

[27] Constitution of the World Health Organization. American journal of public health and the nation’s health. American Journal of Public Health and the Nation’s Health. 1946;36(11):1315–1323. . 10.2105/ajph.36.11.1315 PMC1625885 18016450

[28] Venn JI. On the diagrammatic and mechanical representation of propositions and reasonings. London, Edinburgh. Dublin Philos Mag J Sci. 1880;10(59):1–18.

[29] Gormley GJ, Collins K, Boohan M, et al. Is there a place for e-learning in clinical skills? A survey of undergraduate medical students’ experiences and attitudes. Med Teach. 2009;31(1):e6–12. 19253150 10.1080/01421590802334317

[30] Hammarlund CS, Nilsson MH, Gummesson C. External and internal factors influencing self-directed online learning of physiotherapy undergraduate students in Sweden: a qualitative study. J Educ Eval Health Prof. 2015;12:33. 26101401 10.3352/jeehp.2015.12.33 PMC4536344

[31] Bu F, Steptoe A, Fancourt D. Who is lonely in lockdown? Cross-cohort analyses of predictors of loneliness before and during the COVID-19 pandemic. Public Health. 2020;186:31–34. 32768621 10.1016/j.puhe.2020.06.036 PMC7405905

[32] Pei L, Wu H. Does online learning work better than offline learning in undergraduate medical education? A systematic review and meta-analysis. Med Educ Online. 2019;24(1):1666538. 31526248 10.1080/10872981.2019.1666538 PMC6758693

[33] He M, Tang XQ, Zhang HN, et al. Remote clinical training practice in the neurology internship during the COVID-19 pandemic. Med Educ Online. 2021;26(1):1899642. 33685381 10.1080/10872981.2021.1899642 PMC7946031

[34] Gagnon MP, Legare F, Labrecque M, et al. Perceived barriers to completing an e-learning program on evidence-based medicine. Informatics in primary care. 2007;15(2):83–91. 10.14236/jhi.v15i2.646 17877870

[35] Gardner P, Slater H, Jordan JE, et al. Physiotherapy students’ perspectives of online e-learning for interdisciplinary management of chronic health conditions: a qualitative study. BMC Med Educ. 2016;16(1):62. 26879982 10.1186/s12909-016-0593-5 PMC4754862

[36] Beeckman D, Schoonhoven L, Boucque H, et al. Pressure ulcers: e-learning to improve classification by nurses and nursing students. J Clin Nurs. 2008;17(13):1697–1707. 18592624 10.1111/j.1365-2702.2007.02200.x

[37] Stoddard HA, Borges NJ. A typology of teaching roles and relationships for medical education. Med Teach. 2016;38(3):280–285. 26075952 10.3109/0142159X.2015.1045848

[38] Henry JA, Black S, Gowell M, et al. Covid-19: how to use your time when clinical placements are postponed. BMJ. 2020;369:m1489. 32366500 10.1136/bmj.m1489

[39] Whelan A, Prescott J, Young G, et al. Association of American Medical Colleges: guidance on medical students’ participation in direct in-person patient contact activities Washington DC 2020.

[40] Hawthorne K, Prout H, Kinnersley P, et al. Evaluation of different delivery modes of an interactive e-learning programme for teaching cultural diversity. Patient Educ Couns. 2009;74(1):5–11. 18950978 10.1016/j.pec.2008.07.056

[41] Gensichen JVH, Sönnichsen A, Waldmann U, et al. E-learning for education in primary healthcare- turning the hype into reality: a Delphi study. Eur J Gen Pract. 2009;15(1):11–14. 19353427 10.1080/13814780902864160

[42] Hall AK, Nousiainen MT, Campisi P, et al. Training disrupted: practical tips for supporting competency-based medical education during the COVID-19 pandemic. Med Teach. 2020;42(7):756–761. . 32450049 10.1080/0142159X.2020.1766669

