# Supplementary material for: Undergraduate medical education amid COVID-19: a qualitative analysis of enablers and barriers to acquiring competencies in distant learning using focus groups
Source: Med Educ Online. 2021 Jun 15;26(1):1940765. doi: 10.1080/10872981.2021.1940765 (PMC8208109; doi:10.1080/10872981.2021.1940765)
Supplement: Supplemental Material [file ZMEO_A_1940765_SM1885.zip › Supplementary/Supplement 1 Questioning Routes.docx]

**Supplement 1. Questioning Routes.**

**1. Questioning Route for student focus groups**

Introduction

1. How satisfied have you been with your learning in general this semester?

Motivation

1. How high do you rate your motivation during the digital semester and why?
2. What was motivating for you? What wasn’t?

Learning/Competence Acquisition

Subjective learning success

1. How high do you estimate your learning gain during the digital semester compared to the face-to-face study regarding the study block and practice block and why?
2. What practical skills you need as a doctor do you take away from the digital practice block? What are you missing on your way to becoming a doctor?

**Learner-centered learning**

1. To which extent were you able to set your own focus, implement individual learning styles, and determine an individual pace and depth of learning during the online semester?
2. How flexible did you find the semester in terms of time and location?

**Collaborative learning**

1. Do you have the impression that you learned together and from each other or did you work out things in parallel?

Systematic approach to learning

1. What was different about your learning now this semester? What could be advantages, what could be disadvantages?
2. What resources did you use?
3. Do you feel that you have worked on the cases/content in depth or stayed on the surface?

Structure/implementation

1. What was successful and what was not regarding the digital study block? Why?
2. What was successful and what was not regarding the digital practice block? Why?
3. In the best of all worlds, what would both sections have to look like?

Communication

**Communication with teachers**

1. How did you feel about the communication with your instructors? What was successful and what was not so successful?
2. How did you feel about a camera being turned on by the lecturer?
3. Which way of communication do you think is the best and why? (Live with camera+microphone/microphone only, MS Teams feed, MS Teams chat, email).
4. Can a tutor be a role model in the digital concept?

**Communication with patients**

1. How would you rate the patient contact in the digital format? (Video anamnesis, physical examination) What was different in the communication with the patient via the online format? What are the advantages/disadvantages?
2. Were you able to see the case as a whole? Is something missing?
3. How realistic is the picture you got of the patient via the camera?

**Communication among students**

1. Through which channels did you communicate with your fellow students? Was it more than in the previous semesters or was it similar?
2. Did you discuss content-related or organizational matters with your fellow students?
3. What advantages/disadvantages did this have for you?
4. How do you feel about the connection with your fellow students? Do you feel just as connected to them or is it more distant?

Mental wellbeing

1. How have you been this semester? How healthy did you feel this semester?
2. How high was your stress level in digital learning this semester and why?

Organization

Technology

1. Did you experience any technical problems while participating in the online semester?
2. How do you rate Microsoft Teams as a digital teaching format? Why?

Time management

1. How did the digital teaching format affect your personal time management?
2. Did the digital teaching format give you more time for learning?

Conclusion

1. What is your conclusion about the digital semester in medical school?
2. Is the online semester a blueprint for a new semester?
3. To what extent would you like to see digital formats in your studies in the future? What would you keep? What would you do differently?

**2. Questioning route for teacher focus groups**

Introduction

1. How satisfied were you with your teaching?

Structure/implementation

1. What was rather successful and what was not so successful in the digital study block and why in each case?
2. What was rather successful in the digital practice block via Microsoft Teams and what was not so successful and why in each case?
3. What would a digital practice block week in the best of all worlds look like if resources did not play a role?

Learning/Competence Acquisition

1. Which skills did the students acquire and which not?
2. What are the students missing in the online semester on the way to becoming a doctor and what do they get out of it?
3. How much do you estimate the students' subjective learning gain during the digital semester?
4. Can the physical examination be conveyed via the camera?

Communication

**Communication with students**

1. Where did you find your communication with the students easier or more difficult compared to the classroom semester? What did you find advantageous, what was more of a hindrance?
2. What do you think is the best way to communicate? Live with camera and microphone? Only microphone? MS Teams Chat? E-mail? Why?
3. How important do you think a camera or microphone on is for communication?
4. Can a tutor be a role model in the digital concept?

**Communication between students and patients**

1. How did you feel about student-patient communication in this setting? What were the advantages and disadvantages?
2. Can you learn to build a relationship with the patient online?

Mental wellbeing

1. How healthy do you feel during the digital semester? How do you notice in your teaching and in your contact with students?
2. Has digital teaching affected your stress level? In what way?

Organization

**Technology**

1. Did you experience any technical problems while participating in the online semester?
2. How do you rate Microsoft Teams as a digital teaching format? Why?

**Time management**

1. How has the digital teaching format affected your personal time management?

Conclusion

1. What is your conclusion about the digital semester?
2. Is it a blueprint for future semesters?
3. Are there some things you should keep? Should some things be avoided at all costs?
