# Supplementary material for: Undergraduate medical education amid COVID-19: a qualitative analysis of enablers and barriers to acquiring competencies in distant learning using focus groups
Source: Med Educ Online. 2021 Jun 15;26(1):1940765. doi: 10.1080/10872981.2021.1940765 (PMC8208109; doi:10.1080/10872981.2021.1940765)
Supplement: Supplemental Material [file ZMEO_A_1940765_SM1885.zip › Supplementary/Supplement 2 Results Nonverbal communication.docx]

**Supplement 2. Results – Non-verbal communication.**

**Relief/Positively surprised**

Students and teachers were positively surprised at how well the online semester worked. Word accentuation, head nodding and widened eyes with raised eyebrows underline the astonishment. The positive reactions mostly came promptly and without hesitation. There was unanimity among most participants about this.

**Stress level/mental wellbeing**

Most students felt less stressed during the online semester, had more time for sports and family. For example, study participant 1.2 S emphasizes the words "*significantly more time*", "*sports*" and "*family*". She smiles and leans back in her chair, which seems to reflect her relaxation and joy.

In the second focus group, the participants immediately nod and laugh in agreement when a member talked about mental wellbeing. All participants in this group immediately agree that they were doing very well health wise / mentally in the online semester. Leaning back against the back of the chair and laughing, for example, illustrates the relaxation of participant 2.3 S. here.

In the fourth focus group, the participants are initially hesitant when asked about their mental wellbeing. This express that they have not thought about this yet and thus have not noticed a conscious difference. There is agreement that the stress level in the online semester is not much different from a "*normal*" (4.3 S) semester. The participants laugh and nod to each other.

Study participant 6.2 S already shakes her head while the moderator asks if they felt stressed during the online semester. A reclined sitting position, folded arms and outstretched legs underline his relaxed posture and attitude. The participant slightly shrugs his shoulders several times during his statement while holding his arms folded in front of his chest. This could mean that he cannot imagine that it could have feel differently and that it is clear to him that it was more relaxed.

Some of the teachers felt that the online semester was more stressful. This is illustrated, among other things, by nodding head movements of the speakers and other participants in focus group 3. Gesticulating hand movements underline the statements made. In focus group 5, most participants agreed that it was more relaxed overall. Study participant 5.1 T leans against his chair and keeps a calm tone, the other participants nod in agreement. Study participant 5.2 T expresses that she found it more tiring, laughing at the end and looking at the other participants. This could show her uncertainty as to whether she is the only one in the group with this opinion.

**Motivation**

Motivation was high for most students when they realized that the implementation of the online semester was working. Study participant 4.2 S shrugs her shoulders and shakes her head when she says that it was a "*new situation*", which may represent her not knowing whether it would work before the semester started. Nodding of heads from other participants indicates agreement. Participant 4.4 S makes small hand movements as she says that they were kept "*on the ball*" to illustrate the time or distance and that she was "*more motivated to do it*" the whole time. Study participant 6.1 S also expresses that she was "*much more motivated*". She then pauses to emphasize what she has said. She points to herself with her hand to show that it is her opinion.

**Flexibility**

The overall opinion among students and teachers is that the flexibility in terms of time and place was higher in the online semester and that this is an advantage. Participant 3.1 S emphasizes that he has also been to his "*home*" at times. In doing so, he nods and demonstrates with his hands the different places he has been. In focus group 2, all participants immediately agree that they liked the flexibility in the online semester. They immediately laugh and nod at each other. The flexibility of time and place is also appreciated by the teachers. Participant 3.3 T nods at this and leans forward affirmatively. Again, in focus group 5 in which teachers participated the participants nodded at the statement that one could "*spontaneously postpone an appointment*" (5.1 T). Here, the participant's emphasis is on ‘spontaneously’ and ‘reschedule’. This illustrates that the participant sees the flexible adjustment of appointments as a special feature now in the online semester compared to the face-to-face semester.

**Social contacts/loneliness (students‘ perspective)**

Participant 1.1 S expresses in a regretful tone that "*small talk*" with others was "*lacking*" in the online semester. As she does so, her gaze goes downward. A pause in speech after "*missed*" lends emphasis to the word and illustrates the relevance of the topic from the participant's point of view. Participant 1.3 S from the same group also emphasizes that social contacts had been "*greatly lacking*". He describes his mental state as "*difficult*" (1.3 S), pausing briefly before doing so to look into the distance. This could be interpreted as him searching for the appropriate word, which in turn may show that this state of mind is new and he cannot put it directly into words. Participant 2.3 S answers promptly and without hesitation that it is "*much more distant*". Participant 6.4 S also says that it is not easy to socialize online. She shakes her head at this. In addition, during the summer, "*the social contact in studies*" (6.4 S) was lacking. Her gaze goes downward at this, underscoring her regret.

**Social interaction with students/feedback (teachers’ perspective)**

In both focus groups with teachers, it was expressed that teachers miss the personal social interaction with students. One participant emphasizes that it was "*not the same with a camera*" (3.3 T). He contrasts this with "*face-to-face interaction*" (3.3 T) and uses his hand to illustrate the other person. Participant 5.2 T also expresses that she "*missed the conta*ct". She laughs and leans forward affirmatively. The laughter could be interpreted as nervousness. She looks at the other participants, who nod in agreement.

Furthermore, the teachers express that they get less visual feedback from the students in the online semester. Participant 3.2 T, shrugging her shoulders, emphasizes that there is "*no feedback at all*" and the other participants nod in agreement. Participant 3.4 T then says that it is "*not at all possible to gauge*" whether students are "*bored*" or "*following*". She found this very "*difficult*" (3.4 T). Her voice goes down at this, which could correspond to regret or sadness about it. Participant 5.3 T agrees, describing that the "*process [...] was [missing] in the digital world.*" He nods his head and makes approving hand gestures to his neighbor. He could "*not say for sure*" (5.3 T) what percentage of students "*met the learning objectives*" (5.3 T). The emphasis is on the word 'quite', which underlines the contrast to the face-to-face semester, where one could possibly better assess the learning success of the students.

**Relationship with patients**

Participant 2.3 S emphasizes that she "*found it difficult [to] show empathy via a screen*" in the patient interview. The emphasis on the word 'screen' illustrates the specificity of the online semester and the resulting communication with patients. With her hands, the participant underlines her statement that this was "*super hard*" (2.3 S) and receives nods of agreement from the other participants. She also emphasizes that in one conversation it became "*very emotional*" (3.2 S). She demonstrates with her hands the patient in front of her while describing that he was "crying in front of [the] camera" (3.2 S) and that she could not "*do anything at all*" (3.2 S) due to the distance via camera. She shakes her head and shrugs her shoulders. This shows her helplessness. Her look seems sad. This shows that she regrets not being able to be there for the patient via the camera as she might have been on site. Overall, she describes it as "*not a nice situation*" (3.2 S). Here she laughs nervously, implying that she describes the situation even nicer than it probably actually was.

Participant 4.4 S found it particularly difficult to address "*personal issues such as sexual history*" when communicating with the patient. She explains, "*over the camera [the] threshold was definitely [...] higher*" (4.4 S). She strokes her thigh with her left hand. This could be taken as a nervous gesture reflecting her discomfort in the situation (patient history).

Participant 6.2 S states that communication with the patient in the online semester is "*more of a fact-oriented conversation*" and contrasts this with the "*relationship level with the patient*" that one no longer has online. He sits leaning back in the chair with his arms folded in front of his chest and talks in a matter-of-fact tone, which on the one hand illustrates the fact that the conversation takes place less on an emotional level than the factual level. On the other hand, the reclined sitting position illustrates distance and could be transferred to the distanced communication with the patients.

It is also clear on the part of the teachers that relationship building in patient communication was only possible to a limited extent in the online semester. Participant 3.1 T emphasizes here that relationship building is "*the absolute be-all and end-all*" and that this was "*only possible to a very very very very limited extent now*". The other participants nod at this. In addition, it is difficult to "*comfort*" the patient in digital patient communication (5.2 T). Nodding in agreement from the other participants in focus group 5 and a regretful tone support the participant's point.

**Relationship with teacher (students’ perspective)**

Participant 1.2 S expresses that she "*felt closer to the teachers [...]*". She gives this answer immediately without hesitation. She sits quietly and has her hands folded in her lap. Participant 2.1 S also felt the communication with the teachers in the online semester was "*more personal*". As a reason she mentions the higher resolution of the face, which in a face-to-face lecture can only be seen small in the distance. Here she makes a movement in the air with her hand, which is to demonstrate the teacher in the distance. She shrugs her shoulders as she describes that she "*felt it more personal*" (2.1 p), which might show that she perceives it as a paradox. The other participants from focus group 2 nod in agreement. Participant 6.1 S particularly percieves the use of "*first names*" (6.1 S) in communication with teachers as positive, as it loosens up the atmosphere. She widens her eyes and raises her eyebrows.

**Sense of responsibility/commitment (teachers)**

Participant 3.4 T feels "*more responsible now*" for the students. The emphasis here is on the word 'more responsible'. She is sitting reclined with her legs crossed. She looks at the other participants, nodding. She contrasts the feeling of being responsible with the daily routine on the ward when the students are on site in the clinic and "*everyone [...] is responsible for the weekly student*" (3.4 T). Participant 3.3 T also notes that he "*took more and more intensive time*" in the online semester. He makes a circular hand gesture as he speaks of the students he was "*with*" (3.3 T). He is leaning forward as he does this, which may illustrate his sense of responsibility. In focus group 3, there is agreement that it is "*a different commitment*” (3.1 T) in the online semester to the students. This is evident by participants nodding their heads in agreement.
